# Human-Gut Phages Harbor Sporulation Genes

**DOI:** 10.1128/mbio.00182-23

**Published:** 2023-04-12

**Authors:** Daniel A. Schwartz, Josué A. Rodríguez-Ramos, Michael Shaffer, Rory M. Flynn, Rebecca A. Daly, Kelly C. Wrighton, Jay T. Lennon

**Affiliations:** a Department of Biology, Indiana University, Bloomington, Indiana, USA; b Department of Soil and Crop Sciences, Colorado State University, Fort Collins, Colorado, USA; University of Tennessee at Knoxville

**Keywords:** dormancy, evolution, genomics, guts, metagenomes, microbiome, phage, sporulation

## Abstract

Spore-forming bacteria are prevalent in mammalian guts and have implications for host health and nutrition. The production of dormant spores is thought to play an important role in the colonization, persistence, and transmission of these bacteria. Spore formation also modifies interactions among microorganisms such as infection by phages. Recent studies suggest that phages may counter dormancy-mediated defense through the expression of phage-carried sporulation genes during infection, which can alter the transitions between active and inactive states. By mining genomes and gut-derived metagenomes, we identified sporulation genes that are preferentially carried by phages that infect spore-forming bacteria. These included genes involved in chromosome partitioning, DNA damage repair, and cell wall-associated functions. In addition, phages contained homologs of sporulation-specific transcription factors, notably *spo0A*, the master regulator of sporulation, which could allow phages to control the complex genetic network responsible for spore development. Our findings suggest that phages could influence the formation of bacterial spores with implications for the health of the human gut microbiome, as well as bacterial communities in other environments.

## OBSERVATION

Microbiomes in the human gut are made up of a diverse community of bacteria, archaea, and microeukaryotes, as well as viruses that infect these microorganisms (1). Members of the phylum *Bacillota* (formerly *Firmicutes*) include many spore-forming lineages such as *Bacillus* and *Clostridium*. While spore-forming taxa are indicators of healthy gut microbiomes, some strains within this group are common intestinal pathogens (2, 3). Sporulation is a complex form of dormancy, involving hundreds of genes, that helps these bacteria contend with spatial and temporal variation in environmental conditions in human guts and facilitate transmission (2, 4).

Viruses of microbes, such as bacteriophages, play an important role in shaping gut microbiomes (1). Phage fitness is thought to be enhanced through the encoding of bacterial-like auxiliary metabolic genes (AMGs) that can reprogram and sustain host metabolism during infection (5). The acquisition of other, nonmetabolic genes may allow phages to alter other aspects of bacterial physiology (6). One of the most important determinants of phage fitness is the metabolic activity of the host cell (7, 8). Rates of bacterial metabolism span orders of magnitude, ranging from exponential growth to being nearly inert when cells engage in certain types of dormancy, such as sporulation (9, 10). By entering a state of reduced metabolic activity, microorganisms can defend themselves against phage attack (11, 12), altering selection in ways that could modify coevolutionary dynamics.

Previous work has demonstrated that some phage genomes possess sporulation genes (13–15). In one example, homologs of sporulation-specific sigma factors (*sigG* and *sigF*) were identified in both lytic and lysogenic phages (13). These sigma factors are essential for the developmental transition of a vegetative cell to an endospore (16). When expressed in a host (Bacillus subtilis), the phage-carried sigma factors activate sporulation transcriptional pathways and depress spore yield by up to 99% (13). To date, there has not been any systematic analysis of the prevalence and distribution of sporulation genes in phages. Thus, it remains unknown whether modification of host sporulation is a common phage strategy. In this study, we search for homologs of sporulation genes in genomic and metagenomic data to determine whether phages employ this strategy in human gut microbiomes.

### Identifying sporulation homologs in viral genomes and metagenomes.

We identified sporulation genes in viral genomes and uncultivated viral genomes (UViGs) using DRAM-v (17) (see Fig. S1 and Text S1 in the supplemental material). Specifically, we targeted homologs of well-characterized sporulation genes found in B. subtilis and Clostridioides difficile. We reasoned that phage-carried genes can affect sporulation only if they are in phages that infect a spore-forming host. We therefore designed an enrichment test to identify homologs of sporulation genes that were preferentially found in phages that infect spore-forming hosts. We first evaluated our search strategy by looking for sporulation genes in genomes of phage isolates for which the host was known. Next, we applied the same approach to UViGs assembled from human-gut environments, and for which host predictions had been made in the original studies (18, 19). To minimize the potential for contamination by bacterial sequences, we inspected the annotations of 6,542 gut-derived UViGs in which sporulation genes were detected (Fig. S2), with an average of 117 UViGs inspected per enriched sporulation gene (Fig. S3).

10.1128/mbio.00182-23.2 FIG S1 Workflow for identification of phage homologs of sporulation genes. UViGs, uncultivated virus genomes. Download FIG S1, TIF file, 2.1 MB. Copyright © 2023 Schwartz et al. 2023 Schwartz et al. https://creativecommons.org/licenses/by/4.0/ This content is distributed under the terms of the Creative Commons Attribution 4.0 International license.

10.1128/mbio.00182-23.3 FIG S2 Examples of color-coded genome maps of uncultivated viral genomes (i.e., scaffolds) used in manual curation. Arrows along scaffolds correspond to gene annotations generated by DRAM-v. Gene direction is indicated by arrow direction and vertical position. Gene colors summarize functional annotations of individual genes from KEGG and PFAM. Annotations containing the terms “virion,” “capsid,” “tail,” “terminase,” “baseplate,” “phage,” “virus,” “reverse transcriptase,” or “head” were classified as hallmark viral genes. Genes having an annotation that was not viral or hypothetical were classified as “other annotation.” The sporulation gene of interest (*spo0A* in these examples) is marked by a red arrow with a pink background, while other sporulation genes are marked with cyan or gray background, indicating genes enriched in phages of spore-forming hosts or not, respectively. (a) Rejected, as the *spo0A* gene is found at the edge of the scaffold. (b) Rejected; scaffold region with *spo0A* has many nonviral annotated genes and a high frequency of changes in gene direction, both indicating this is a bacterial genome fragment. The right-hand side of the scaffold appears to be viral based on the presence of multiple hallmark viral genes. (c and d) Accepted; in both cases *spo0A* is nested between hallmark viral genes in scaffolds that otherwise contain many genes lacking annotation and few switches of gene direction. As needed, the raw annotation files for a scaffold were also inspected. The viral gene annotations were added manually in these plots to illustrate that. Scaffold labels on the left indicate dataset name, scaffold index within dataset (indexed separately for each gene), and VirSorter category (NA, not available for these scaffolds). Download FIG S2, TIF file, 6.3 MB. Copyright © 2023 Schwartz et al. 2023 Schwartz et al. https://creativecommons.org/licenses/by/4.0/ This content is distributed under the terms of the Creative Commons Attribution 4.0 International license.

10.1128/mbio.00182-23.4 FIG S3 Manual inspection of gut-derived uncultivated viral genomes (i.e., scaffolds) containing sporulation genes from references 13 and 14. In each panel the data are separated between genes for which a few examples of true viral origin were observed (“likely viral”), never observed (“unlikely viral”) or observed in very few instance (<5 scaffolds; “possible viral”). (a) The number of scaffolds in which each of the sporulation genes was detected and the number of scaffolds manually inspected. (b) The fraction of the inspected scaffolds in which the focal sporulation gene is likely of viral origin (“true”), likely originates from bacterial contamination (“false”), or is difficult to determine (“maybe”). Download FIG S3, TIF file, 6.5 MB. Copyright © 2023 Schwartz et al. 2023 Schwartz et al. https://creativecommons.org/licenses/by/4.0/ This content is distributed under the terms of the Creative Commons Attribution 4.0 International license.

### Phages possess nonregulatory genes required for sporulation.

**FIG 1:**
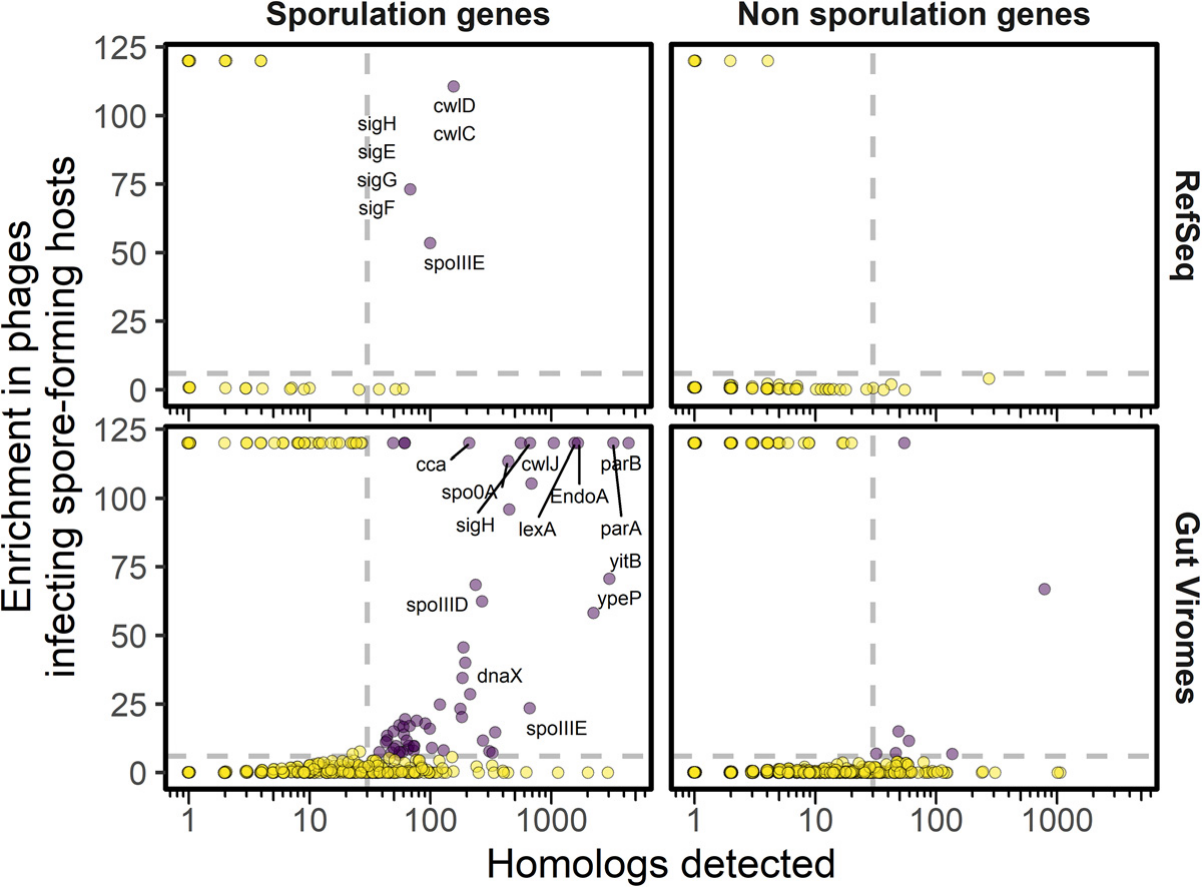
Sporulation genes are enriched in phages that infect spore-forming bacteria. Homologs of both sporulation and nonsporulation genes were identified in RefSeq isolate phage genomes and in published gut viromes (18, 19) using DRAM-v. However, genes enriched among phages infecting spore-forming hosts (purple points) were mostly sporulation-related genes. The number of homologs detected for each gene is plotted on the *x* axis. For each of the genes, a hypergeometric enrichment test evaluated if it was found in phages that infect spore-forming hosts more than the random expectation given the number of phages infecting spore-forming hosts among all phages surveyed. The enrichment result [–log_10_ (hypergeometric *P* value)] is plotted on the *y* axis. Purple points represent enriched genes with an adjusted *P* value of <10^−6^ (horizontal dashed line) and a sample size of >30 (vertical dashed line). Representative names of B. subtilis sporulation genes are provided for genes that were enriched and of viral origin.

Our search identified 31 phage-carried homologs of sporulation genes (Table 1). These sporulation genes were enriched in phages that infect spore-forming hosts (Fig. 1). Many of the phage-carried homologs were nonregulatory genes involved in an assortment of sporulation-related processes such as chromosome partitioning, DNA damage repair, and cell wall-associated functions (Table 1). The acquisition of these genes might allow phages to promote or impede specific steps of spore development or its eventual germination. For example, phages may use chromosome segregation genes to increase the probability of entrapment (and survival) of the phage genome in the spore during the asymmetric division separating the developing spore from the mother cell (20). Alternatively, it is possible that some of these genes are used by phages for functions other than sporulation. Chromosome segregation genes are known to be used by phages that establish extrachromosomal, plasmid-like lysogeny (21). Likewise, cell wall hydrolases used by the host to restructure the cell during sporulation (*cwlJ*, *sleB*, *spoIID*) could be repurposed as endolysins to burst the host cell at the completion of the phage lytic cycle (22). Further experimental investigation will be required to establish the phage functions of the sporulation gene homologs that we have catalogued in this work.

**TABLE 1:** Sporulation genes detected in viral genomes and metagenomes^*a*^

KO^*b*^	Locus no.^*c*^	Gene^*c*^	Type^*d*^	Function^*e*^
K01356	BSU_17850	*lexA*	R	*lexA* repressor
K03086	BSU_25200	*sigA*	R	RNA polymerase sigma factor RpoD
K03091	BSU_00980	*SigH*	R	RNA polymerase sigma-H factor
	BSU_15320	*SigE*	R	RNA polymerase sigma-E factor
	BSU_15330	*SigG*	R	RNA polymerase sigma-G factor
	BSU_23450	*sigF*	R	RNA polymerase sigma-F factor
	CD630_07720	*SigF*	R	RNA polymerase sigma-F factor
	CD630_12300	*SigK*	R	RNA polymerase sigma-K factor
	CD630_26420	*SigG*	R	RNA polymerase sigma-G factor
	CD630_26430	*sigE*	R	RNA polymerase sigma-E factor
K04769	BSU_560	*spoVT*	R	Stage V sporulation protein T
	CD630_34990	*spoVT*	R	Stage V sporulation protein T
K06283	BSU_36420	*spoIIID*	R	Stage III sporulation protein D
	CD630_1260	*spoIIID*	R	Stage III sporulation protein D
K06284	BSU_370	*abrB*	R	Transition state regulatory protein AbrB
K07699	BSU_24220	*spo0A*	R	Stage 0 sporulation protein A
	CD630_12140	*spo0A*	R	Stage 0 sporulation protein A
K07738	CD630_26400	*nrdR*	R	Transcriptional regulator, repressor NrdR family
K03496	BSU_40970	*parA*	R+NR	Sporulation initiation inhibitor protein Soj
	CD630_36720	*soj*	R+NR	Transcriptional regulator, sporulation initiation inhibitor, chromosome partitioning protein
K00390	BSU_10930	*yitB*	NR	Phosphoadenosine phosphosulfate reductase
K00640	CD630_15950	*cysE*	NR	Serine acetyltransferase (SAT)
K00820	CD630_1200	*glmS*	NR	Glucosamine-fructose-6-phosphate aminotransferase (isomerizing)
K00974	BSU_22450	*cca*	NR	CCA-adding enzyme
K01142	BSU_40880	*exoA*	NR	Exodeoxyribonuclease, repair of oxidative DNA damage in spores
K01449	BSU_02600	*CwlJ*	Nr	Cell wall hydrolase CwlJ
	BSU_22930	*sleB*	NR	Spore cortex-lytic enzyme
	CD630_35630	*NA*	NR	Putative spore cortex-lytic hydrolase
K02049	BSU_30610	*ytlC*	NR	ABC transporter ATP-binding protein
K02343	CD630_160	*dnaX*	NR	DNA polymerase III subunits gamma and tau
K03466	BSU_16800	*spoIIIE*	NR	Spore DNA translocase
K03497	BSU_40960	*parB*	NR	Stage 0 sporulation protein J
	CD630_36710	*spo0J*	NR	Stage 0 sporulation protein J, site-specific DNA-binding protein
K03657	CD630_7490	*NA*	NR	Putative DNA helicase, UvrD/REP type
K03664	BSU_33600	*smpB*	NR	SsrA-binding protein
K03698	BSU_9930	*yhaM*	NR	3′–5′ exoribonuclease yhaM
K06381	BSU_36750	*spoIID*	NR	Stage II sporulation protein D
	CD630_1240	*spoIID*	NR	Stage II sporulation protein D
K06412	BSU_490	*spoVG*	NR	Septation protein SpoVG
	CD630_35160	*spoVG*	NR	Regulator required for spore cortex synthesis
K07171	CD630_34610	*EndoA*	NR	Endoribonuclease toxin
K10716	BSU_31322	*yugO*	NR	Potassium channel protein YugO
K10979	BSU_13410	*ykoV*	NR	DNA repair protein YkoV
K014487	BSU_17410	*cwlC*	NR	Mother cell lysis
	BSU_01530	*cwlD*	NR	Spore cortex peptidoglycan synthesis
K02647	BSU_28670	*ysfB*	U	Hypothetical protein; similar to carbohydrate diacid transcriptional activator
K03469	BSU_21970	*ypeP*	U	Hypothetical protein; similar to RNase HI
K07175	BSU_14810	*ylaK*	U	Hypothetical protein; similar to PhoH

a These genes were enriched in phages infecting spore-forming hosts and were validated by manual inspection of annotations to have a viral origin.

b KO, KEGG ortholog identifier.

c “Locus no.” and “gene” refer to the gene locus number and name of the sporulation gene(s) associated with a KO. BSU loci are from Bacillus subtilis (KEGG taxon T00010), and CD630 loci are from Clostridioides difficile (KEGG taxon T00487).

d Locus type reflects whether a KO is a regulatory gene (R), a nonregulatory gene (NR), or a hypothetical or uncharacterized function (U).

e The “Function” column provides a description of the KO from SubtiWiki for B. subtilis or KEGG for *C. difficle*.

### Phages also possess genes that regulate sporulation.

Nearly one-third of the sporulation homologs (*n* = 9) identified in phage genomes and metagenomes are transcriptional regulators (Table 1). This finding is different than most examples of AMGs, where the phages control a metabolic process by phage-carried enzymes or by expression of modulators of host enzyme activity (5). It may be that phages manipulate host sporulation by interfering with the tightly regulated transcriptional program that is essential for this complex developmental process (2). Such findings are consistent with recent experimental findings regarding sporulation-specific sigma factors, where the ectopic expression of phage-carried *sigG* and *sigF* homologs altered the transcriptional program of B. subtilis, resulting in reduced spore yield (13).

**FIG 2:**
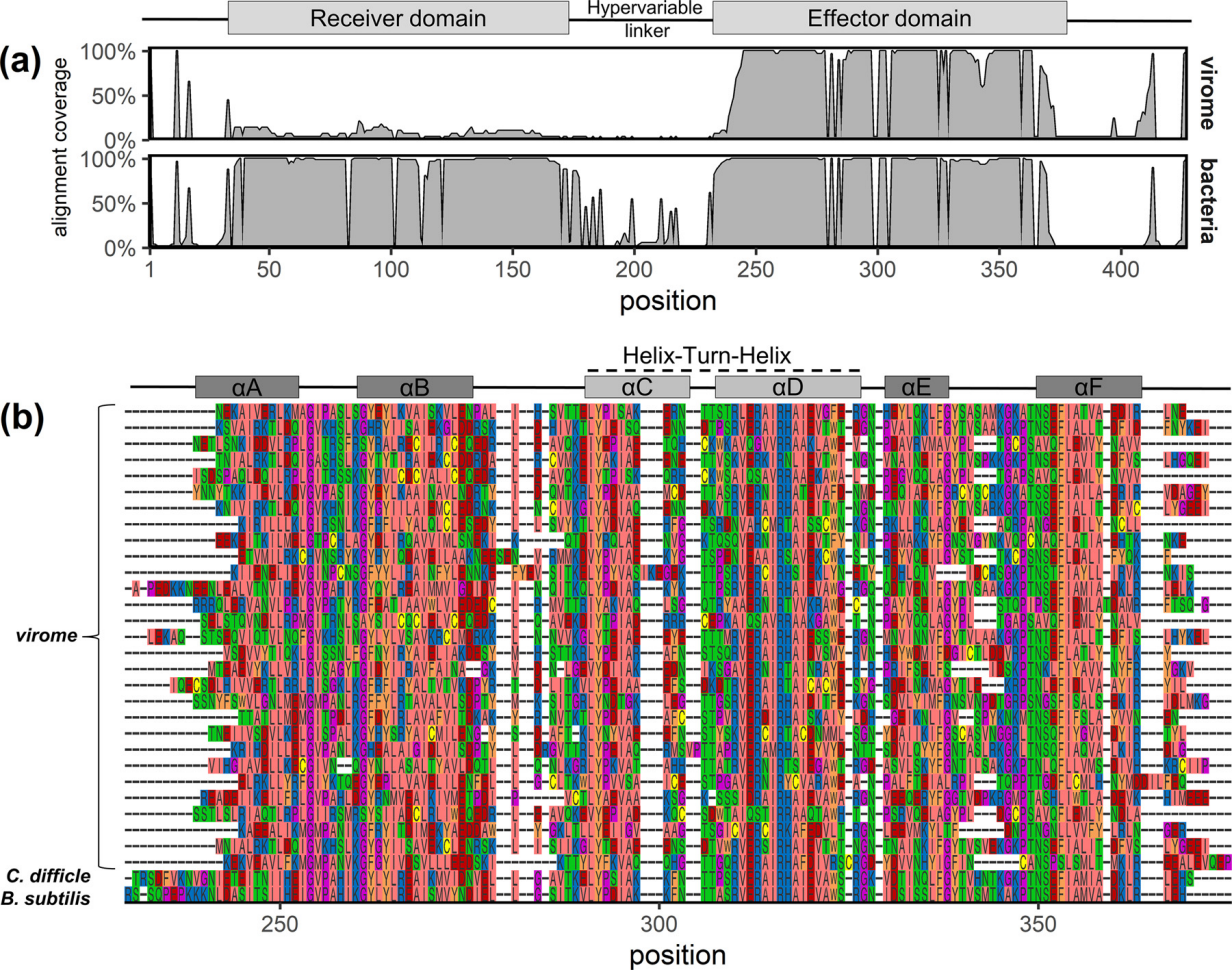
Multiple sequence alignment of protein sequences of *spo0A* homologs from viromes (*n* = 29) and diverse bacteria (*n* = 57; from COG5801). (a) Summary of alignment coverage showing the percentage of nongap characters at each position, separated by the source of the sequence. *Spo0A* from viromes align to the C-terminal effector domain of the bacterial genes. (b) Focus on effector domain, showing all virome sequences aligned with *spo0A* of model spore-forming bacteria Clostridioides difficile and Bacillus subtilis. The alpha helices of the effector domain are indicated above the sequences. Sequence colors correspond to physicochemical properties of amino acids, using the Zappo coloring scheme. Information on functional and structural domains is from reference 26.

Most notable among phage-carried regulators are homologs of *spo0A*, the master regulator of sporulation initiation that is conserved among all spore-forming bacteria (4). Interestingly, the homologs found in phages are truncated versions of *spo0A* that contain the DNA-binding effector domain, but not the receiver domain (Fig. 2). The latter is responsible for modifying the DNA-binding activity in response to environmental and physiological signals received via the phosphorelay signal-transduction system (23). The truncation suggests that phage-carried *spo0A* may not require the normal host signals to activate or repress the initiation of host sporulation (24). In addition to transcriptional regulators, phage genes included other potential posttranscriptional regulators (RNA binding *spoVG*, and translation-related genes *cca* and *smpB*). Taken together, the bioinformatic findings here and laboratory results (13) suggest some phages may overcome dormancy defenses by targeting the regulation of sporulation. Compared to the use of nonregulatory genes, this is likely to be a more efficient strategy for altering the course of a complex cellular program.

### Phage-carried sporulation genes occur in diverse environments.

The recovery of phage-carried sporulation genes is not restricted to the human gut. We identified sporulation genes in UViGs originating from diverse environments (Table S1). Of the 30 sporulation genes identified in gut-derived UViGs, we found 23 that also occur in phages from terrestrial and aquatic environments (Fig. S4). Thus, phage manipulation of sporulation may be a common phenomenon in environments where spore-forming bacteria are found.

10.1128/mbio.00182-23.1 TABLE S1 Viromes analyzed in this study. Download Table S1, DOCX file, 0.06 MB. Copyright © 2023 Schwartz et al. 2023 Schwartz et al. https://creativecommons.org/licenses/by/4.0/ This content is distributed under the terms of the Creative Commons Attribution 4.0 International license.

10.1128/mbio.00182-23.5 FIG S4 Sporulation genes detected in uncultivated viral genomes (=scaffolds) from diverse ecosystems. “Host-associated” ecosystem includes samples derived from human guts, but these are different from the data sets discussed in the main text. The number of scaffolds in which a gene was detected by DRAM-v and validated by manual inspection of scaffold annotations is shown. Empty cells indicate no scaffolds detected. See Text S1 for details on methods and samples. Download FIG S4, TIF file, 2.0 MB. Copyright © 2023 Schwartz et al. 2023 Schwartz et al. https://creativecommons.org/licenses/by/4.0/ This content is distributed under the terms of the Creative Commons Attribution 4.0 International license.

### Implications and future directions.

Sporulation is an ancient, complex, and important trait that contributes to the persistence and transmission of beneficial and pathogenic members of the mammalian gut microbiome. While sporulation can reduce virus infection, our analysis supports the view that phages may use host-like genes to overcome this host defense mechanism (25). Specifically, our study provides genomic and metagenomic evidence that phages carry homologs of multiple sporulation genes, which may influence the transition of bacteria between active and dormant states in host-associated and environmental ecosystems. The evolutionary drivers and ecological consequences of phage-carried sporulation genes remain to be investigated (15). Our work demonstrates how partitioning phages by a specific host trait (e.g., sporulation) can be used to identify genes used by phages to influence the same host trait.

### Data availability.

The data products of this study, including sequence data for sporulation genes identified in UViGs, are available at https://doi.org/10.5281/zenodo.7742410. All code used in this study is available at https://github.com/LennonLab/spore_amg and in the Zenodo repository linked above.

10.1128/mbio.00182-23.6 TEXT S1 Details on methods. Download Text S1, DOCX file, 0.03 MB. Copyright © 2023 Schwartz et al. 2023 Schwartz et al. https://creativecommons.org/licenses/by/4.0/ This content is distributed under the terms of the Creative Commons Attribution 4.0 International license.
