# Supplementary figures and images for: Human-Gut Phages Harbor Sporulation Genes
Source: mBio. 2023 Apr 12;14(3):e00182-23. doi: 10.1128/mbio.00182-23 (PMC10294663; doi:10.1128/mbio.00182-23)

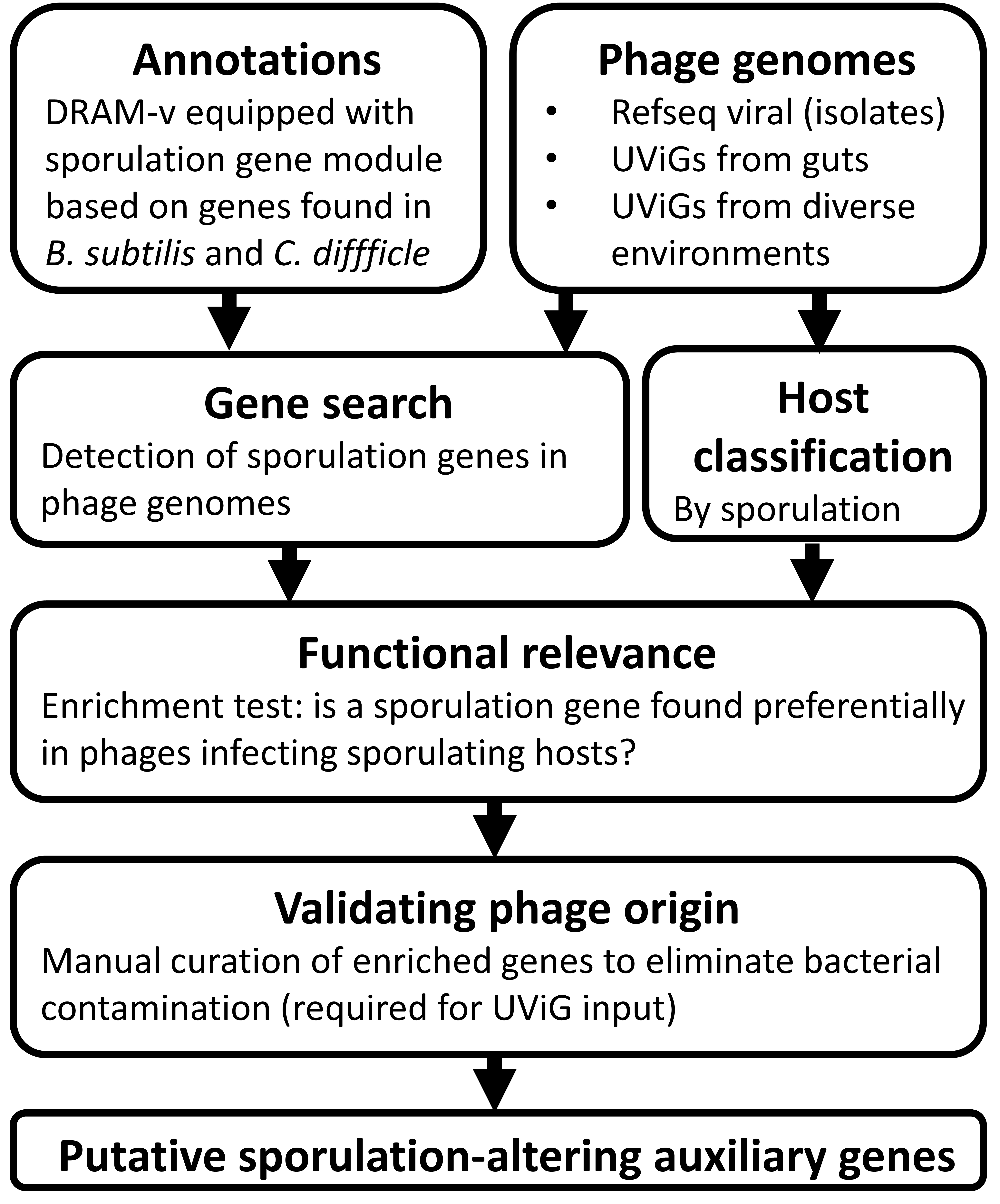

Supplement: FIG S1 [file mbio.00182-23-s0002.tif]

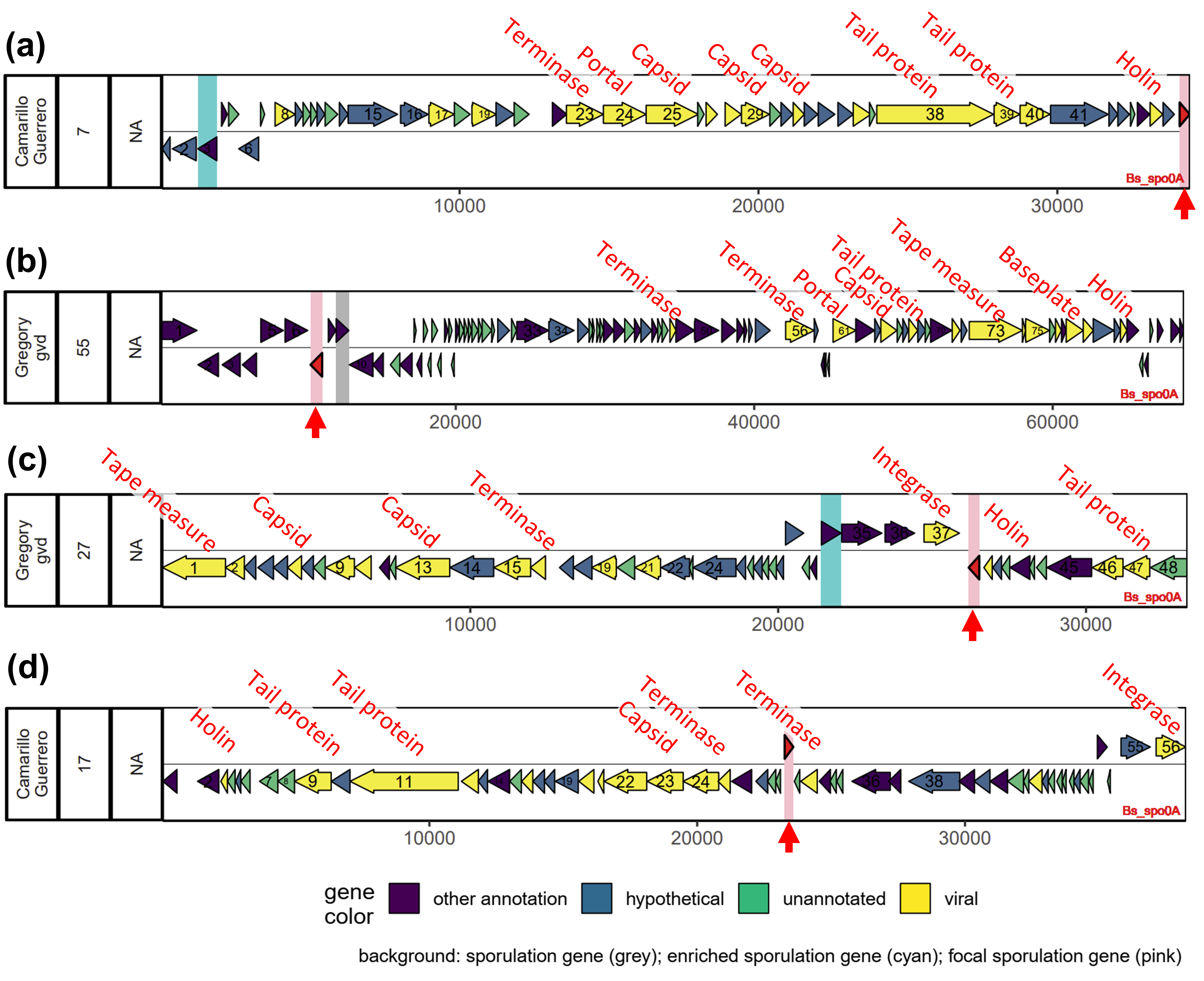

Supplement: FIG S2 [file mbio.00182-23-s0003.tif]

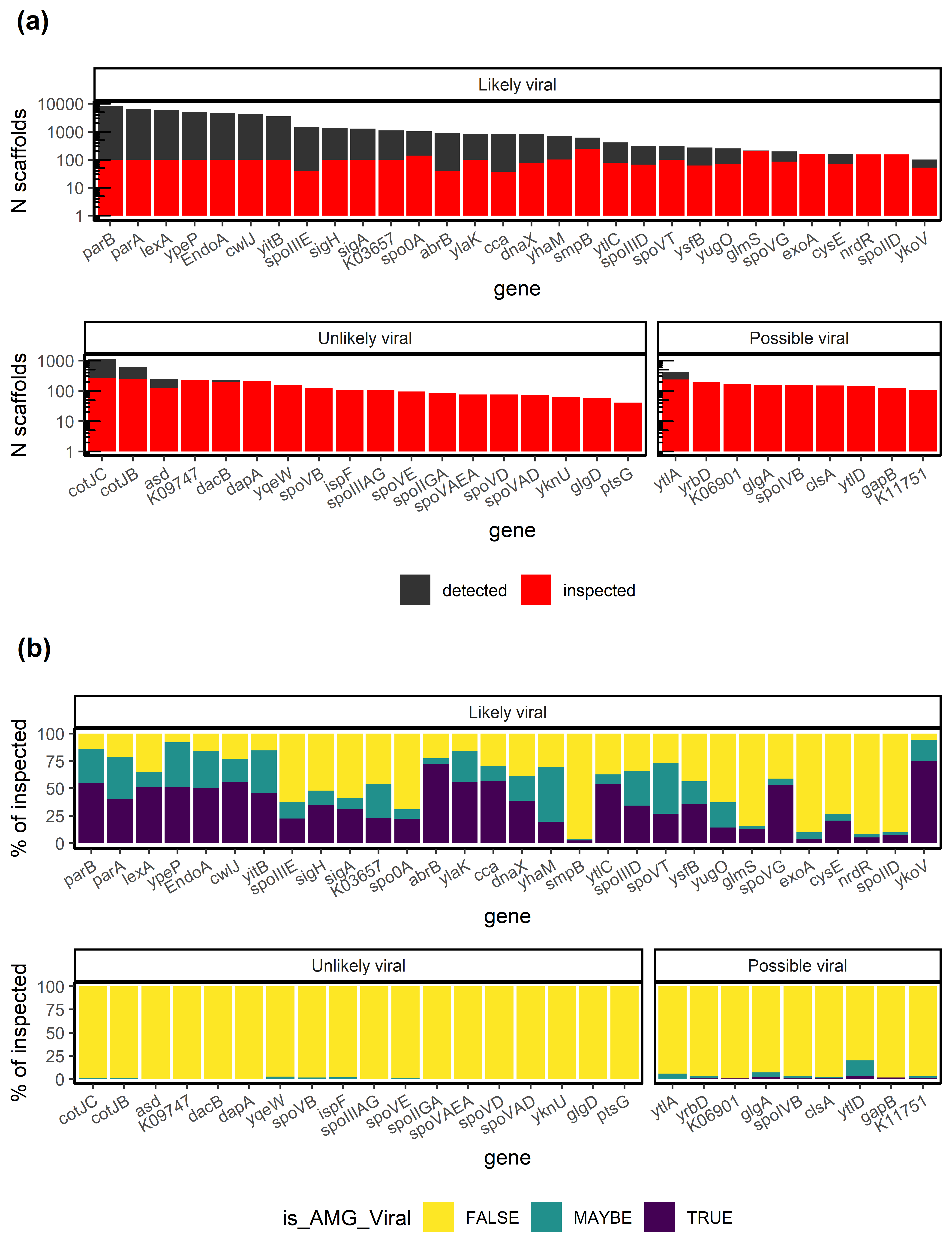

Supplement: FIG S3 [file mbio.00182-23-s0004.tif]

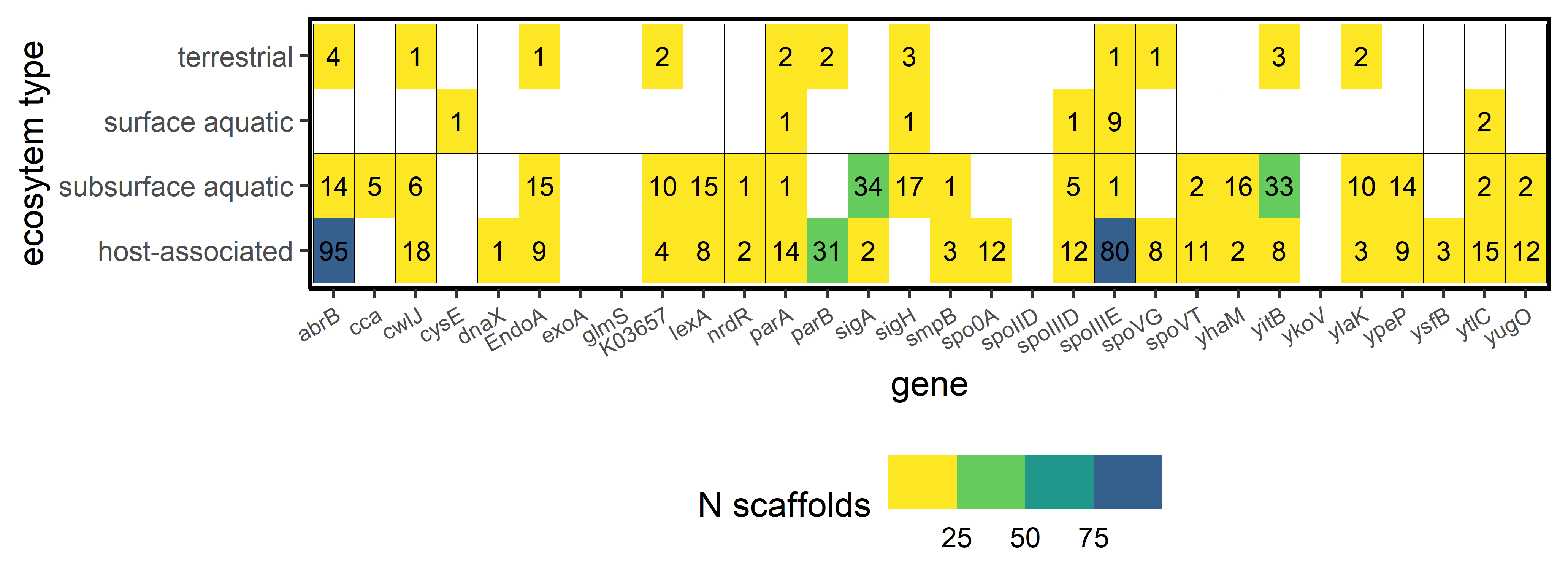

Supplement: FIG S4 [file mbio.00182-23-s0005.tif]
