# Supplementary material for: Human-Gut Phages Harbor Sporulation Genes
Source: mBio. 2023 Apr 12;14(3):e00182-23. doi: 10.1128/mbio.00182-23 (PMC10294663; doi:10.1128/mbio.00182-23)
Supplement: TABLE S1 [file mbio.00182-23-s0006.docx]

| **Set name** | **Ecosystem type** | **Description** | **Source** |
| --- | --- | --- | --- |
| Gut Virome Database | host-associated | human gut study | (1) |
| Gut Phage Database | host-associated | human gut study | (2) |
| Anaerobic_Digestors | host-associated | incubations using sludge from wastewater treatment plant in Ft Collins, CO, digesting food waste from CSU | Wrighton, Unpublished |
| ARDEC_agriculture | terrestrial | soil samples from CSU's Agricultural Research, Development and Education Center (ARDEC) under a variety of cover crops | Wrighton, Unpublished |
| Columbia_River | surface aquatic | sediment samples from Columbia River, WA | (3) |
| CT_feces | host-associated | incubation of human feces (from methylamines study) amended with purified condensed tannin | Wrighton, unpublished |
| CT_soil | terrestrial | incubation of wetland soil (from OWC study) amended with purified condensed tannin | (4) |
| East_River | surface aquatic | sediment and pore water samples from East River, CO | (5) |
| Fire_soils | terrestrial | soil samples across a wildfire burn grandient in northern CO and southern WY | (6) |
| Frac_DJ_basin | subsurface aquatic | fracking wells in Colorado (unpublished data) | Wrighton, Unpublished |
| Frack_STACK | subsurface aquatic | fracking wells in Oklahoma | (7) |
| Fractured_shale | subsurface aquatic | Fracking wells in Appalachian Basin | (8) |
| Human_Microbiome_Project | host-associated | human gut study | these were described in this paper (9) |
| Huttenhower | host-associated | human gut study, reads were from this dataset were assembled (unpublished) | (10) |
| Lake_Yojoa | surface aquatic | water column samples from Lake Yojoa, Honduras | Wrighton, Unpublished |
| Methylamines | host-associated | human fecal samples from healthy adults (from Cincinnati OH) | (11) |
| Moose_rumen | host-associated | rumen fluid samples from a moose in Alaska | (12) |
| Ohio_groundwater | subsurface aquatic | groundwater samples from 3 sites in OH | (13) |
| OWC_wetlands | surface aquatic | samples from wetland on shore of Lake Erie, CO | (14) |
| Prado_wetlands | surface aquatic | water samples from a constructed wetland in CA | (15) |
| Prairie_potholes | surface aquatic | sediment samples from Prairie Pothole region (lakes) in SD | (16) |
| Salmonella_chow | host-associated | feces from mice with and without Salmonella infection (fed normal mouse chow) | (17) |
| Salmonella_HFD | host-associated | feces from mice with and without Salmonella infection (fed a High Fat Diet) | Wrighton, Unpublished |
| Stordalen_mire | terrestrial | soil samples across a permafrost thaw gradient from Stordaline Mire Sweden (published data, viral-centric paper) | (18) |

1. Gregory AC, Zablocki O, Zayed AA, Howell A, Bolduc B, Sullivan MB. 2020. The gut virome database reveals age-dependent patterns of virome diversity in the human gut. Cell Host & Microbe 28:724-740. e8.

2. Camarillo-Guerrero LF, Almeida A, Rangel-Pineros G, Finn RD, Lawley TD. 2021. Massive expansion of human gut bacteriophage diversity. Cell 184:1098-1109. e9.

3. Rodríguez-Ramos JA, Borton MA, McGivern BB, Smith GJ, Solden LM, Shaffer M, Daly RA, Purvine SO, Nicora CD, Eder EK. 2022. Genome-resolved metaproteomics decodes the microbial and viral contributions to coupled carbon and nitrogen cycling in river sediments. bioRxiv.

4. McGivern BB, Tfaily MM, Borton MA, Kosina SM, Daly RA, Nicora CD, Purvine SO, Wong AR, Lipton MS, Hoyt DW. 2021. Decrypting bacterial polyphenol metabolism in an anoxic wetland soil. Nature Communications 12:1-16.

5. Saup C, Bryant S, Nelson A, Harris K, Sawyer A, Christensen J, Tfaily M, Williams K, Wilkins M. 2019. Hyporheic zone microbiome assembly is linked to dynamic water mixing patterns in snowmelt‐dominated headwater catchments. Journal of Geophysical Research: Biogeosciences 124:3269-3280.

6. Nelson AR, Narrowe AB, Rhoades CC, Fegel TS, Daly RA, Roth HK, Chu RK, Amundson KK, Young RB, Steindorff AS. 2022. Wildfire-dependent changes in soil microbiome diversity and function. Nature Microbiology 7:1419-1430.

7. Amundson KK, Borton MA, Daly RA, Hoyt DW, Wong A, Eder E, Moore J, Wunch K, Wrighton KC, Wilkins MJ. 2022. Microbial colonization and persistence in deep fractured shales is guided by metabolic exchanges and viral predation. Microbiome 10:1-15.

8. Daly RA, Roux S, Borton MA, Morgan DM, Johnston MD, Booker AE, Hoyt DW, Meulia T, Wolfe RA, Hanson AJ. 2019. Viruses control dominant bacteria colonizing the terrestrial deep biosphere after hydraulic fracturing. Nature Microbiology 4:352-361.

9. Shaffer M, Borton MA, McGivern BB, Zayed AA, La Rosa SL, Solden LM, Liu P, Narrowe AB, Rodríguez-Ramos J, Bolduc B. 2020. DRAM for distilling microbial metabolism to automate the curation of microbiome function. Nucleic Acids Research 48:8883-8900.

10. Lloyd-Price J, Arze C, Ananthakrishnan AN, Schirmer M, Avila-Pacheco J, Poon TW, Andrews E, Ajami NJ, Bonham KS, Brislawn CJ. 2019. Multi-omics of the gut microbial ecosystem in inflammatory bowel diseases. Nature 569:655-662.

11. Borton MA, Shaffer M, Hoyt DW, Jiang R, Ellenbogen J, Purvine S, Nicora CD, Eder EK, Wong AR, Smulian AG, Lipton MS, Krzycki JA, Wrighton KC. 2022. Targeted curation of the gut microbial gene content modulating human cardiovascular disease. bioRxiv doi:10.1101/2022.06.20.496735:2022.06.20.496735.

12. Solden LM, Naas AE, Roux S, Daly RA, Collins WB, Nicora CD, Purvine SO, Hoyt DW, Schückel J, Jørgensen B. 2018. Interspecies cross-feeding orchestrates carbon degradation in the rumen ecosystem. Nature Microbiology 3:1274-1284.

13. Danczak R, Johnston M, Kenah C, Slattery M, Wrighton KC, Wilkins MJ. 2017. Members of the Candidate Phyla Radiation are functionally differentiated by carbon-and nitrogen-cycling capabilities. Microbiome 5:1-14.

14. Angle JC, Morin TH, Solden LM, Narrowe AB, Smith GJ, Borton MA, Rey-Sanchez C, Daly RA, Mirfenderesgi G, Hoyt DW. 2017. Methanogenesis in oxygenated soils is a substantial fraction of wetland methane emissions. Nature Communications 8:1-9.

15. Reilly JF, Horne AJ, Miller CD. 1999. Nitrate removal from a drinking water supply with large free-surface constructed wetlands prior to groundwater recharge. Ecological Engineering 14:33-47.

16. Dalcin Martins P, Danczak RE, Roux S, Frank J, Borton MA, Wolfe RA, Burris MN, Wilkins MJ. 2018. Viral and metabolic controls on high rates of microbial sulfur and carbon cycling in wetland ecosystems. Microbiome 6:1-17.

17. Leleiwi I, Rodriguez-Ramos J, Shaffer M, Sabag-Daigle A, Kokkinias K, Flynn RM, Daly RA, Kop LF, Solden LM, Ahmer BMM, Borton MA, Wrighton KC. 2022. Exposing new taxonomic variation with inflammation: A murine model-specific genome database for gut microbiome researchers. bioRxiv doi:10.1101/2022.10.24.513540:2022.10.24.513540.

18. Emerson JB, Roux S, Brum JR, Bolduc B, Woodcroft BJ, Jang HB, Singleton CM, Solden LM, Naas AE, Boyd JA. 2018. Host-linked soil viral ecology along a permafrost thaw gradient. Nature Microbiology 3:870-880.
