# Supplementary material for: Human-Gut Phages Harbor Sporulation Genes
Source: mBio. 2023 Apr 12;14(3):e00182-23. doi: 10.1128/mbio.00182-23 (PMC10294663; doi:10.1128/mbio.00182-23)
Supplement: TEXT S1 [file mbio.00182-23-s0001.docx]

**Human-gut phages harbor sporulation genes**

Daniel A. Schwartz^1^, Josué A. Rodríguez-Ramos^2^, Michael Shaffer^2^, Rory M. Flynn ^2^, Rebecca A. Daly^2^, Kelly C. Wrighton^2^, Jay T. Lennon^1^

^1^ Department of Biology, Indiana University, Bloomington, IN 47405

^2^ Department of Soil and Crop Sciences, Colorado State University, Fort Collins, CO 80521

* Corresponding authors: danschw@iu.edu and lennonj@iu.edu

# **SUPPLEMENTARY TEXT**

**List of sporulation genes —** To search for sporulation genes in phages, we compiled a list of sporulation-related genes for the two best studied endospore-forming bacteria: *Bacillus subtilis* and *Clostridioides* *difficile*. For *B. subtilis*, we identified 880 sporulation genes that have been experimentally evaluated and summarized in the Subtiwiki database (1) and other publications (2, 3). For *C. difficile*, we identified a total of 350 sporulation genes. We included genes that were functionally annotated as being involved in sporulation (GenBank: CP016318.1) along with sporulation genes that were identified in at least two of four curated studies (2, 4-6). We mapped sporulation genes for both species to functional orthologous groups in the KEGG Orthology (KO) database using the KEGGREST R Package (7), which enabled detection of diverse sporulation gene homologs using hidden-Markov-model (HMM) based annotations by DRAM (see below).

**Classification of host sporulation capacity —** To evaluate if a phage’s host is likely to be a spore-former, we compiled data on spore formation within families of the phylum Bacillota (formerly Firmicutes). Assuming that only members of the Bacillota form endospores, we assigned members of all other phyla as non-sporulators. Within the Bacillota we combined data on spore-formation from two previous studies (8, 9). We classified families as spore-formers if >50% of the species identified by Browne et al. (9) were found to be spore formers. For 10 families whose spore-forming status differed between the two studies (8, 9), we assigned spore-forming status by manual inspection of the underlying data in these studies, supplemented by additional sources (10-12). Within 402 families from the Bacillota defined by the genome taxonomy database (GTDB v202), we assigned 165 as spore-formers and 92 as non-spore-formers, while 145 were not assigned due to insufficient data.

**Identification of UViGs, manual curation of genomes, and detection of sporulation genes in phages** **—** Publicly available datasets used in this study were downloaded from their respective repositories with their predicted host information (13, 14) and annotated using DRAM-v v1.3.1 to identify candidate homologs of sporulation genes (15). DRAM is a program that annotates genomes and assigns the genes to predefined metabolic modules. Using KO IDs corresponding to the curated list of sporulation genes, we supplemented DRAM with a sporulation module, which is publicly available (<https://github.com/WrightonLabCSU/sporulation_utils>). As previously described, DRAM’s viral mode (DRAM-v) classifies metabolic genes in viral genomes as putative auxiliary metabolic genes (AMGs). It then provides a score of 1-5 that describes confidence metrics to annotation calls as well as distillate flags that aid in distinguishing between bacterial genes that originate from viral genomes and those from bacterial genome fragments erroneously classified as viral. For publicly available datasets that did not have available viral contigs, we downloaded the respective assemblies (Table S1) and then used VirSorter v1.0.3 with default settings to identify uncultivated viral genomes (UViGs) from our metagenomics datasets (16). We selected high confidence UViGs that were ≥ 10kb in size and were assigned to categories 1-2 and 4-5 by VirSorter. These were then clustered into UViGs at 95% ANI across 85% of the shortest contig using ClusterGenomes 5.1 (<https://github.com/simroux/ClusterGenomes>) according to established standards (17). We then used DRAM-v v1.3.1 with default settings for functional annotation.

To avoid misidentification of bacterial contaminants as bacterial genes encoded by viruses (i.e., AMGs), we supplemented our discovery pipeline with manual inspection and curation of UViGs (18, 19). For each of the sporulation genes enriched in the analysis of gut viromes, we performed a manual inspection of large samples of annotated UViGs containing those genes by plotting the genome maps with features of the DRAM annotations (Fig. S2). We only considered genes as good hits if they corresponded to high-confidence AMG categories 1-3 as suggested by DRAM-v default settings (15). Briefly, putative virus-encoded sporulation genes were considered truly viral if they were nested between viral hallmark genes (annotations containing the words "virion", "capsid", "tail", "terminase", "baseplate", "phage", "virus", "reverse transcriptase" or "head") and genes with no annotation (including hypothetical genes and genes of unknown function). We also excluded sporulation genes that were at or near the edge of a scaffold (DRAM AMG flag “F”). Finally, we excluded sporulation genes that were found in regions having characteristics of bacterial origin such as regions in which most genes were annotated as non-viral by KEGG or PFAM in the DRAM annotations, regions being indicative of bacterial transposons with no viral annotations (DRAM AMG Flag “T”), or regions having many gene direction switches. Sporulation genes that were detected >10 times in clearly viral contig regions are considered likely viral sporulation genes.

*Enrichment of sporulation genes in viral isolates* – We reasoned that phage-encoded genes can only affect sporulation if they are encoded by phages that infect a host capable of forming spores. We first searched for sporulation genes in phage genomes from the RefSeq viral database (v202). This is a database curated and annotated by the National Center for Biotechnology Information (NCBI) with many genomes of phage isolates, so there is little concern of bacterial contamination in the sequences, and the identity of the hosts is known. We conducted the proof-of-concept test using only *B. subtilis* sporulation genes. Phage hosts identified using the virus-host database (20) were classified as spore-formers or non-spore-formers based on family-level taxonomy, as described above. We were able to classify host sporulation capacity of 3,650 phages, of which 257 are known to infect spore-forming hosts. For each candidate gene, we conducted a hypergeometric enrichment test to determine if the gene was detected in phages infecting spore-formers more than expected by a random draw of phages from the pool of 3,650 phages. *P*-values were adjusted for multiple comparisons using the Benjamini-Hochberg method (21). Sporulation genes with less than 30 observations were excluded from our analysis.

*Enrichment of sporulation genes in viral metagenomes by host sporulation –* To expand our search of viral sporulation genes beyond phage isolates, we applied the same enrichment approach described above using two previously published datasets of UViGs assembled from human gut samples. Typically, there is a high relative abundance of spore-forming Bacillota in these environments, which was confirmed by applying our spore-forming classification to host predictions that were made as part of the original studies (13, 14). We annotated the UViGs from these two datasets using DRAM-v equipped with the full sporulation module based on sporulation genes from both *B. subtilis* and *C. difficile*. We matched the available host predictions (published alongside these datasets) to our list of sporulators at the family level. We classified host sporulation capacity of 53,624 UViGs, of which 25,630 are predicted to infect spore-forming hosts. Enrichment tests for sporulation genes found in viral contigs of both datasets were conducted as described above. In addition to the human gut samples, we used DRAM-v equipped with the full sporulation module to annotate UViGs from a variety of metagenomic datasets originating from aquatic, terrestrial and host associated environments (Table S1).

**Multiple sequence alignment of viral and bacterial *spo0A* genes —** To compare viral and bacterial homologs of spo0A we retrieved bacterial *spo0A* protein sequences from the Clusters of Orthologous Genes (COG) database (COG5801 in v2020 (22). We clustered the sequences using CD-HIT-est (23) at 65% identity over 90% of the largest sequence (-c 0.65 -aS 0.9). Bacterial sequences were aligned together with sequences from UViGs that passed manual inspection (see above) using MUSCLE v5 (24). Multiple sequence alignments were visualized using the ggmsa (25) package in R (26).

**Code and data availability —** Data products of this study, including sequence data for sporulation genes identifies in UViGs are available at <https://doi.org/10.5281/zenodo.7742410>. All code used in this study is available at <https://github.com/LennonLab/spore_amg>, and in the Zenodo repository linked above.

# **SUPPLEMENTARY REFERENCES**

1. Pedreira T, Elfmann C, Stülke J. 2022. The current state of SubtiWiki, the database for the model organism *Bacillus subtilis*. Nucleic Acids Research 50:D875-D882.

2. Ramos-Silva P, Serrano M, Henriques AO. 2019. From root to tips: sporulation evolution and specialization in *Bacillus subtilis* and the intestinal pathogen *Clostridioides difficile*. Molecular Biology andEevolution 36:2714-2736.

3. Weller C, Wu M. 2015. A generation-time effect on the rate of molecular evolution in bacteria. Evolution 69:643-652.

4. Dembek M, Barquist L, Boinett CJ, Cain AK, Mayho M, Lawley TD, Fairweather NF, Fagan RP. 2015. High-throughput analysis of gene essentiality and sporulation in *Clostridium difficile*. mBio 6:e02383-14.

5. Fimlaid KA, Bond JP, Schutz KC, Putnam EE, Leung JM, Lawley TD, Shen A. 2013. Global analysis of the sporulation pathway of *Clostridium difficile*. PLoS Genetics 9:e1003660.

6. Saujet L, Pereira FC, Serrano M, Soutourina O, Monot M, Shelyakin PV, Gelfand MS, Dupuy B, Henriques AO, Martin-Verstraete I. 2013. Genome-wide analysis of cell type-specific gene transcription during spore formation in *Clostridium difficile*. PLoS Genetics 9:e1003756.

7. Tenenbaum D, Maintainer BP. 2022. KEGGREST: Client-side REST access to the Kyoto Encyclopedia of Genes and Genomes (KEGG), vR package version 1.36.3.

8. Galperin MY. 2013. Genome Diversity of Spore-Forming Firmicutes. Microbiology Spectrum 1:1.2.09.

9. Browne HP, Forster SC, Anonye BO, Kumar N, Neville BA, Stares MD, Goulding D, Lawley TD. 2016. Culturing of ‘unculturable’human microbiota reveals novel taxa and extensive sporulation. Nature 533:543-546.

10. Rosenberg E, DeLong EF, Lory S, Stackebrandt E, Thompson F. 2014. The Prokaryotes: Firmicutes and Tenericutes. Springer.

11. Vos P, Garrity G, Jones D, Krieg NR, Ludwig W, Rainey FA, Schleifer K-H, Whitman WB. 2011. Bergey's manual of systematic bacteriology: Volume 3: The Firmicutes, vol 3. Springer Science & Business Media.

12. Johnson CN, Whitehead TR, Cotta MA, Rhoades RE, Lawson PA. 2014. Peptoniphilus stercorisuis sp. nov., isolated from a swine manure storage tank and description of Peptoniphilaceae fam. nov. International Journal of Systematic and Evolutionary Microbiology 64:3538-3545.

13. Camarillo-Guerrero LF, Almeida A, Rangel-Pineros G, Finn RD, Lawley TD. 2021. Massive expansion of human gut bacteriophage diversity. Cell 184:1098-1109. e9.

14. Gregory AC, Zablocki O, Zayed AA, Howell A, Bolduc B, Sullivan MB. 2020. The gut virome database reveals age-dependent patterns of virome diversity in the human gut. Cell Host & Microbe 28:724-740. e8.

15. Shaffer M, Borton MA, McGivern BB, Zayed AA, La Rosa SL, Solden LM, Liu P, Narrowe AB, Rodríguez-Ramos J, Bolduc B. 2020. DRAM for distilling microbial metabolism to automate the curation of microbiome function. Nucleic Acids Research 48:8883-8900.

16. Roux S, Enault F, Hurwitz BL, Sullivan MB. 2015. VirSorter: mining viral signal from microbial genomic data. PeerJ 3:e985.

17. Roux S, Adriaenssens EM, Dutilh BE, Koonin EV, Kropinski AM, Krupovic M, Kuhn JH, Lavigne R, Brister JR, Varsani A. 2019. Minimum information about an uncultivated virus genome (MIUViG). Nature Biotechnology 37:29-37.

18. Pratama AA, Bolduc B, Zayed AA, Zhong Z-P, Guo J, Vik DR, Gazitúa MC, Wainaina JM, Roux S, Sullivan MB. 2021. Expanding standards in viromics: in silico evaluation of dsDNA viral genome identification, classification, and auxiliary metabolic gene curation. PeerJ 9:e11447.

19. Guo J, Vik D, Pratama AA, Roux S, Sullivan MB. 2021. Viral sequence identification SOP with VirSorter2 V.3 doi:dx.doi.org/10.17504/protocols.io.bwm5pc86, protocol.io.

20. Mihara T, Nishimura Y, Shimizu Y, Nishiyama H, Yoshikawa G, Uehara H, Hingamp P, Goto S, Ogata H. 2016. Linking virus genomes with host taxonomy. Viruses 8:66.

21. Benjamini Y, Hochberg Y. 1995. Controlling the false discovery rate: a practical and powerful approach to multiple testing. Journal of the Royal statistical society: series B (Methodological) 57:289-300.

22. Galperin MY, Wolf YI, Makarova KS, Vera Alvarez R, Landsman D, Koonin EV. 2021. COG database update: focus on microbial diversity, model organisms, and widespread pathogens. Nucleic acids research 49:D274-D281.

23. Li W, Godzik A. 2006. Cd-hit: a fast program for clustering and comparing large sets of protein or nucleotide sequences. Bioinformatics 22:1658-1659.

24. Edgar RC. 2022. High-accuracy alignment ensembles enable unbiased assessments of sequence homology and phylogeny. bioRxiv doi:10.1101/2021.06.20.449169:2021.06.20.449169.

25. Zhou L, Feng T, Xu S, Gao F, Lam TT, Wang Q, Wu T, Huang H, Zhan L, Li L. 2022. ggmsa: a visual exploration tool for multiple sequence alignment and associated data. Briefings in Bioinformatics.

26. R Core Team. 2021. R: A Language and Environment for Statistical Computing, R Foundation for Statistical Computing, Vienna, Austria. <https://www.R-project.org/>.
